# Synergistic effects of α‐synuclein, tau, and amyloid pathology on mitophagy in dementia with Lewy bodies

**DOI:** 10.1002/alz.092955

**Published:** 2025-01-03

**Authors:** Xu Hou, Tyrique Richardson, Fabienne C Fiesel, Michael G. Heckman, Shunsuke Koga, Dennis W. Dickson, Wolfdieter Springer

**Affiliations:** ^1^ Mayo Clinic, Jacksonville, FL USA

## Abstract

**Background:**

Directed by the enzyme pair PINK1 and PRKN, mitophagy is a crucial mitochondrial quality control mechanism that selectively decorates damaged mitochondria with phosphorylated ubiquitin (pS65‐Ub), facilitating their lysosomal degradation. The dynamic pS65‐Ub signal accumulates upon enhanced activation from increased mitochondrial damage or upon reduced autophagic‐lysosomal flux. Previous studies including ours demonstrated altered mitophagy and elevated pS65‐Ub levels in Parkinson’s and Alzheimer’s disease brains that also independently associated with α‐synuclein, tau, or amyloid pathology. However, their combined impact on mitophagy and organelle function remains unclear. Notably these three pathologies often coexist in susceptible brain areas of a neurodegenerative condition named dementia with Lewy bodies (DLB). We herein investigated the interactions of α‐synuclein, tau, and amyloid pathology to uncover their combined effects on mitophagy in a large DLB cohort.

**Method:**

This study included 371 DLB autopsy brains with neurologically normal cases as controls. Immunohistochemistry for the mitophagy marker pS65‐Ub in the hippocampus and amygdala was performed and associations of pS65‐Ub levels with neuropathological measures from the same region were evaluated. Immunofluorescence co‐staining of pS65‐Ub, phospho‐α‐synuclein, and phospho‐tau was conducted to study their interactions at single‐cell level.

**Result:**

pS65‐Ub positive cells were significantly more abundant in the hippocampus and amygdala of DLB cases compared to controls. Significant associations were observed between pS65‐Ub levels with age, brain weight, Braak stage, Thal phase as well as counts of Lewy body (LB), neurofibrillary tangle (NFT), and senile plaque (SP) in both regions. Strong synergistic effects of LB and NFT pathologies on pS65‐Ub accumulation were found in the amygdala, while only additive effects of LB and SP pathologies were observed. Single‐cell analysis in the amygdala showed that most affected cells contained either LB or NFT inclusion rather than co‐residing two pathologies within the same cell. Notably, cells harboring LB pathology exhibited mainly smaller, granular pS65‐Ub deposits (type G), possibly associated with increased mitochondrial dysfunction. Conversely, those containing NFT showed larger, vacuolar pS65‐Ub deposits (type V), likely resulting from reduced lysosomal degradation.

**Conclusion:**

Our study revealed complex interactions of α‐synuclein, tau, and amyloid pathology on mitophagy alteration in DLB brains, highlighting different molecular mechanisms underlying α‐synuclein‐ and tau‐associated mitophagy alterations.

